# Transversus Abdominis Plane Catheter Bolus Analgesia after Major Abdominal Surgery

**DOI:** 10.1155/2012/596536

**Published:** 2012-05-16

**Authors:** Nils Bjerregaard, Lone Nikolajsen, Thomas Fichtner Bendtsen, Bodil Steen Rasmussen

**Affiliations:** ^1^Department of Anesthesiology, Aalborg Hospital, Aarhus University, 9000 Aalborg, Denmark; ^2^Department of Anesthesiology, Aarhus University Hospital, 8000 Aarhus, Denmark; ^3^Danish Pain Research Center, Aarhus University Hospital, 8000 Aarhus, Denmark

## Abstract

*Purpose*. Transversus abdominis plane (TAP) blocks have been shown to reduce pain and opioid requirements after abdominal surgery. The aim of the present case series was to demonstrate the use of TAP catheter injections of bupivacaine after major abdominal surgery. *Methods*. Fifteen patients scheduled for open colonic resection surgery were included. After induction of anesthesia, bilateral TAP catheters were placed, and all patients received a bolus dose of 20 mL bupivacaine 2.5 mg/mL with epinephrine 5 **μ**g/mL through each catheter. Additional bolus doses were injected bilaterally 12, 24, and 36 hrs after the first injections. Supplemental pain treatment consisted of paracetamol, ibuprofen, and gabapentin. Intravenous morphine was used as rescue analgesic. Postoperative pain was rated on a numeric rating scale (NRS, 0–10) at regular predefined intervals after surgery, and consumption of intravenous morphine was recorded. *Results*. The TAP catheters were placed without any technical difficulties. NRS scores were ≤3 at rest and ≤5 during cough at 4, 8, 12, 18, 24, and 36 hrs after surgery. Cumulative consumption of intravenous morphine was 28 (23–48) mg (median, IQR) within the first 48 postoperative hours. *Conclusion*. TAP catheter bolus injections can be used to prolong analgesia after major abdominal surgery.

## 1. Introduction

Epidural analgesia is commonly used for the treatment of postoperative pain after major abdominal surgery despite the well-known risks and the long list of contraindications [1, 2]. During the last few years, interest has grown concerning the use of transversus abdominis plane (TAP) block as an alternative to epidural analgesia. A TAP block provides analgesia of the anterolateral abdominal wall through blockade of the lateral and anterior cutaneous branches of Th7 to L1 as shown in volunteers by McDonnell et al. [3]. Clinical trials have shown that a bilateral single-shot TAP block reduces pain after large bowel resection and total abdominal hysterectomy [4, 5]. 

The duration of a single-shot TAP block is limited by the pharmacokinetics of the local anesthetic used, and therefore, the use of TAP catheters has been described in order to prolong the effect of the TAP block through infusion or injection of local anesthetic [6–10]. Only two prospective, randomized studies have been carried out [11, 12]. Kadam and Field [11] randomized 40 patients undergoing non-specified major abdominal surgery to receive either a single-shot TAP block at the end of surgery followed by a 72 hr infusion at 8–10 mL/hr of 0.2% ropivacaine 2 mg/mL and fentanyl patient-controlled analgesia (PCA) (TAP group, *n* = 20) or fentanyl PCA only (control group, *n* = 20). Pain scores and consumption of fentanyl were significantly lower in the TAP group on the first and second days after surgery. Niraj et al. [12] randomized 62 patients undergoing major hepatobiliary or renal surgery to receive either intermittent bolus injections of 1 mg/kg bupivacaine 3.75 mg/mL every 8 hr via subcostal TAP catheters placed at the end of surgery (TAP group, *n* = 29) or an epidural infusion of bupivacaine 1.25 mg/mL and fentanyl 2 *μ*g/mL (epidural group, *n* = 33). There were no significant differences in visual analog pain scores (VAS, 0–10) between the epidural group and the TAP group during coughing at 8 hr (VAS = 4.0 (2.5, 5.3 [0–8.5]) and 4.0 (2.3, 6.0 [0–7.5]), resp.) and at 72 hr (VAS = 2.5 (1.0, 5.0 [0–6.0]) and 2.0 (0.8, 4.0 [0–5.0]), resp.). Values are median (IQR (range)).

So far, no trials have examined the analgesic effect of intermittent bolus injections via TAP catheters placed preoperatively using the posterior approach for major abdominal surgery. The purpose of the present case series was to demonstrate the use of intermittent bolus injections of bupivacaine through bilateral TAP catheters as part of a multimodal analgesic regimen in the first 48 hours after open colonic resection surgery.

## 2. Materials and Methods

The case series was registered at http://www.clinicaltrials.gov/, ID: NCT01395043, and approved by the regional ethical committee. Fifteen patients undergoing elective lower major abdominal surgery with laparotomy and colon resection were prospectively included. As this was a case series, only registration of the patients accepting to participate was done. Enrolment started in September 2010 and finished in June 2011. Written informed consent was obtained before enrollment. Primary exclusion criteria were allergies to morphine or bupivacaine or inability to provide informed consent. Secondary exclusion criteria were reoperation within the first 48 hours or postoperative mechanical ventilation.

General anesthesia was induced with propofol 1-2 mg/kg or thiopenthal 3–5 mg/kg, remifentanil 1 *μ*g/kg and suxamethonium 1 mg/kg or cisatracurium 0.15 mg/kg at the discretion of the attending anesthesiologist. Following endotracheal intubation, anesthesia was maintained with sevoflurane at MAC 1 and remifentanil 0.3–1.0 *μ*g/kg/min. An intravenous dose of fentanyl 1-2 *μ*g/kg was given at the end of surgery. After induction of anesthesia, TAP catheters were placed bilaterally as described below. Surgery was performed by trained surgeons with all incisions performed in the lower abdominal wall below the umbilicus.

The TAP catheters were placed by the same experienced anesthetist under sterile conditions. A linear high-frequency ultrasound probe (HFL38, 13–6 MHz) covered with a sterile sheath (SITE-RITE* Probe Cover kit, Bard Access Systems Inc, Salt Lake City, USA) was used. An 18-gauge Touhy needle (Perican, B. Braun, Melsungen AG, Melsungen, Germany) was advanced in plane in a medial to lateral direction with ultrasound guidance using a SonoSite S-Nerve (SonoSite, Bothell, WA, USA) apparatus. The point of insertion was between the anterior and the mid-axillary line and between the lower costal margin and the iliac crest, based on the best visualization of TAP, expected surgical incision and preoperative stoma site marking. After reaching the TAP with the Touhy needle, hydrodissection was done with 10 mL of isotonic saline. An epidural catheter (Braun Perifix catheter, B. Braun, Melsungen AG, Melsungen, Germany) was introduced through the Touhy needle. With the Touhy needle bevel facing posteriorly, the catheter was advanced 15–20 cm inside the TAP in order to avoid displacement during patient movement and ambulation. After hydrodissection the advancement of the TAP catheter was unproblematic, although advancing the catheter less than 15 cm would probably have been sufficient to avoid displacement. The Touhy needle was removed and a filter (Perifix Filter 0.2 *μ*m, B. Braun, Melsungen AG, Melsungen, Germany) was connected to the catheter. Twenty mL of bupivacaine 2.5 mg/mL with epinephrine 5 *μ*g/mL was injected via each catheter with direct real-time ultrasound visualization to ensure correct placement of the TAP catheter. The TAP catheters were fixed using an epidural plaster (EPI-FIX, Unomedical Ltd, Stonehouse, Great Britain) and Mefix self-adhesive fixation (Mölnlycke, Health Care AB, Gothenburg, Sweden).

Three additional bolus doses of 20 mL bupivacaine 2.5 mg/mL were injected bilaterally via the TAP catheters 12, 24, and 36 hr after the first bolus dose (i.e., if duration of surgery was 2 hr, the second, third, and fourth bolus doses were given 10, 22, and 34 hr after end of surgery, resp.). In addition, all patients received a postoperative multimodal analgesic regimen consisting of paracetamol 1000 mg every 6 hr, ibuprofen 400 mg every 8 hr, and gabapentin 400 mg every 8 hr daily. Intravenous (IV) morphine 5–10 mg was used as rescue medication with the aim of ensuring a pain intensity of ≤3 at rest and ≤5 during coughing on a NRS, 0–10. Postoperative pain is generally considered to be acceptable if pain intensity is kept below these NRS levels.

Intensity of pain was assessed 0, 1, 2, 4, 8, 12, 18, 24, and 36 hr after surgery. Cumulative consumption of morphine within the first 48 postoperative hours was also recorded.

## 3. Results

Fifteen patients, 7 males and 8 females, aged 54 to 80 years, were included in the study (see Table 1 for baseline characteristics). All patients received bilateral TAP catheters after induction of anesthesia. Six patients underwent extensive surgery due to infiltration of the primary cancer leading to further resection of nearby tissue such as pelvic floor, urinary bladder, gastric ventricle, and liver. None of the patients underwent reoperation or mechanical ventilation within the first 48 hr.

**Figure 1:**
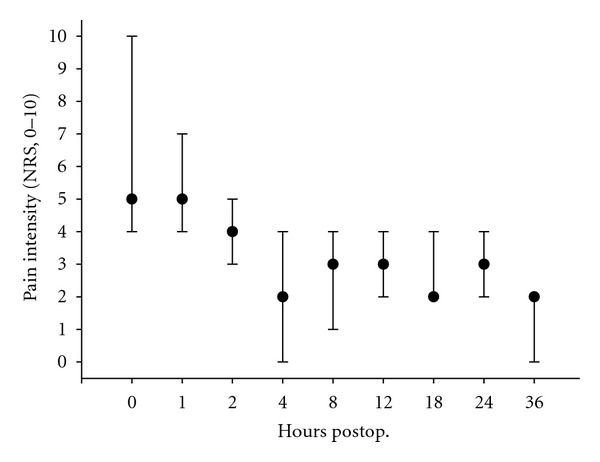
Intensity of pain at rest. Intensity of pain was assessed on a numerical rating scale (0–10) 0, 1, 2, 4, 8, 12, 18, 24, 36 hr after end of surgery. Data are expressed as median (interquartile range). *n* = 15 (*n* = 11 and *n* = 14 after 12 and 18 hr, resp.).

**Figure 2:**
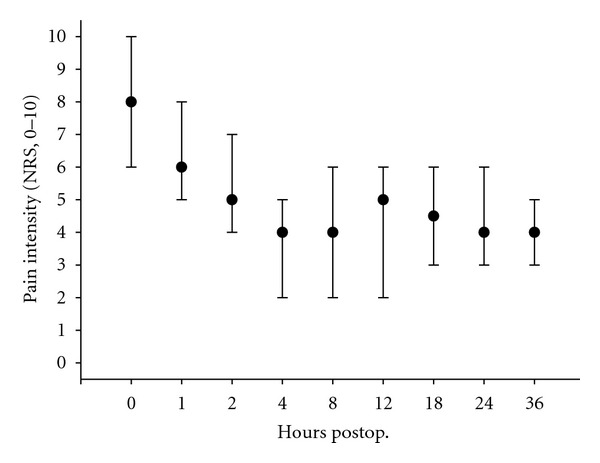
Intensity of pain during coughing. Intensity of pain was assessed on a numerical rating scale (0–10) 0, 1, 2, 4, 8, 12, 18, 24, 36 hr after end of surgery. Data are expressed as median (interquartile range). *n* = 15 (*n* = 11 and *n* = 14 after 12 and 18 hr, resp.).

As can be seen from Figures 1 and 2, the median NRS scores at rest and during coughing were ≤3 and ≤5, respectively, except for the first 2 postoperative hours. The high NRS scores at 0, 1, and 2 hr were primarily due to high scores among the patients who underwent extensive surgery.

The cumulative consumption of IV morphine was 28 (23–48) mg (median, IQR) within the first 48 postoperative hours. Patients who underwent less extensive surgery (*n* = 9) consumed 23 (21–28) mg (median, IQR) IV morphine, whereas patients who underwent extensive surgery (*n* = 6) consumed 50 (34–60) mg (median, IQR) IV morphine. Morphine was administrated intravenously, except for two patients who received a total of 6 doses of oral morphine. In those cases a 1 : 3 ratio was used for conversion from oral to IV morphine.

All patients, except one who was severely walking-impaired before surgery, were mobilized and walked on the day of surgery. No complications, infections, or systemic side effects to bupivacaine were observed during the 48 hr study period. In two patients, one of the TAP catheters was accidentally pulled out during mobilization on the second postoperative day and hence the fourth dose of bupivacaine was only given in the remaining catheter. In another patient, one of the TAP catheters was removed before surgery on request of the surgeon due to close proximity to the surgical field. The catheter was replaced immediately after surgery.

## 4. Discussion

Ultrasound guided TAP block is a relatively new technique and data on the efficacy of TAP block for abdominal analgesia are sparse and conflicting [13–17]. Very limited data describe the use and effect of TAP catheters in order to prolong the analgesic effect of TAP block by continuous infusion or repeated bolus injections of local analgesics. In the present case series, bolus injections were used in order to achieve repeated hydrodissection of the TAP and a significant spread of local anesthetic in the entire TAP. We showed that administration of repeated bolus doses of bupivacaine as part of a multimodal analgesic regimen resulted in acceptable pain-scores and relatively low opioid requirements, comparable to what has been found in other studies [11, 12]. 

The present case series also demonstrates the importance of a careful selection of patients when choosing this technique for postoperative analgesia. The patients with extensive surgery required more morphine and had high levels of pain during the first 2 postoperative hours. The TAP block only generates analgesia of the anterolateral abdominal wall extending to the anterior axillary line; hence, there is no analgesic effect to cover the pelvic floor, visceral pain, retroperitoneum, or the abdominal wall posterior to the anterior axillary line. The moderate or even poor pain control during the first two hours postoperatively was largely caused by poor analgesic effect in the subgroup of patient who underwent extensive surgery. As the TAP catheter has no effect on visceral pain or pain deriving from the pelvis floor, we would expect the TAP catheters to be insufficient to that type of surgery. 

The systemic effect of local analgesics is known to reduce postoperative pain, [18–20] and the plasma concentration of local analgesics used for TAP block has been shown to reach considerable levels with the commonly used catheter dosages [21, 22]. Plasma levels of bupivacaine were not measured in the present study. With a total dose of 100 mg bupivacaine every 12 hr, a robust safety margin concerning toxic effect was secured. However, it is unknown whether the systemic effect of the bupivacaine administered had any effect on pain levels or opioid requirements.

Some practical issues must be considered. Placing a TAP catheter can be done with the patient in the supine position and with less concern for coagulopathy and hemodynamics compared to the placement of an epidural catheter. The posterior insertion permits preoperative placement, as the catheter is kept away from the surgical field. Preoperative placement is preferable as drapes, tissue oedema, intraabdominal air, and surgical drains may hamper the ultrasonographic visualization of the TAP. Also, a joint effort must be made with the surgeons and nurses to reduce the risk of soiling the TAP-dressing, accidental pulling or dislodgement of the catheter during placement, or removal of the surgical dressing. Visualization of the Touhy needle and the epidural-type catheter with ultrasound can be difficult with the equipment used in the present study. However, more echogenic needles and catheters are already on the market and they may facilitate both visualization and placement of a TAP catheter.

We acknowledge several limitations of the present case series. First, it is not randomized or blinded. Second, only a limited number of patients was included. Third, the dermatome level of the block was not registered. Even when these limitations are taken into consideration, intermittent bolus injections of bupivacaine via TAP catheters seem to be a promising alternative to epidural analgesia. However, randomized, double-blind trials are necessary in order to evaluate the efficacy of TAP catheter analgesia.

## 5. Conclusion

TAP catheter bolus injections can be used after major abdominal surgery as part of a multimodal analgesic regimen. The technique is probably best suited for non-extensive surgery where the pain is derived primarily from the abdominal wall incision. This case series presents the use of ultrasound-guided bilateral transversus abdominis plane catheters, placed using the posterior approach, with intermittent bolus injections of bupivacaine, as part of a multimodal analgesic regimen after major abdominal surgery.

## Figures and Tables

**Table 1:** Baseline characteristics (*n* = 15).

Gender (M/F)	7/8
Age (yr)	66 (54–81)
ASA	
I	3
II	6
III	6
Body mass index	27.0 (21.1–36.4)
Duration of surgery (min)	130 (65–240)
Blood loss during surgery (mL)	200 (25–2200)
Comorbidity* (*n*)	13
Arterial hypertension	6
Other cardiac disease	2
Chronic obstructive pulmonary disease	2
Previous stroke	3
Diabetes	1
Other comorbidity^†^	9
Preoperative consumption of opioids (*n*)	3

*5 patients had one comorbidity and 8 patients had two or more comorbidities.

^†^Other co-morbidity included myxoedema, arthritis, benign prostatic hyperplasia, former deep venous thrombosis, dyspepsia, and previous pulmonary tuberculosis.

Values are median (range), otherwise absolute number of cases recorded. ASA = American Society of Anesthesiologists.
